# Correlations between iodine uptake, invasive CT features and pleural invasion in adenocarcinomas with pleural contact

**DOI:** 10.1038/s41598-023-43504-0

**Published:** 2023-09-27

**Authors:** Yingdong Chen, Qianwen Huang, Hua Zhong, Anqi Li, Zeyang Lin, Xiaoxi Guo

**Affiliations:** 1 Department of the Radiology, Zhongshan Hospital, Medicine School, Xiamen University, Xiamen, 361004 China; 2 Department of the Pathology, Zhongshan Hospital, Medicine School, Xiamen University, Xiamen, 361004 China

**Keywords:** Lung cancer, Image processing

## Abstract

Pleural contact in lung cancers does not always imply pleural invasion (PI). This study was designed to determine whether specific invasive CT characteristics or iodine uptake can aid in the prediction of PI. The sample population comprised patients with resected solid lung adenocarcinomas between April 2019 and May 2022. All participants underwent a contrast enhanced spectral CT scan. Two proficient radiologists independently evaluated the CT features and iodine uptake. Logistic regression analyses were employed to identify predictors for PI, via CT features and iodine uptake. To validate the improved diagnostic efficiency, accuracy analysis and ROC curves were subsequently used. A two-tailed *P* value of less than 0.05 was considered statistically significant. We enrolled 97 consecutive patients (mean age, 61.8 years ± 10; 48 females) in our study. The binomial logistic regression model revealed that a contact length > 10 mm (OR 4.80, 95% CI 1.92, 11.99, *p *= 0.001), and spiculation sign (OR 2.71, 95% CI 1.08, 6.79, *p *= 0.033) were independent predictors of PI, while iodine uptake was not. Enhanced sensitivity (90%) and a greater area under the curve (0.73) were achieved by integrating the two aforementioned CT features in predicting PI. We concluded that the combination of contact length > 10 mm and spiculation sign can enhance the diagnostic performance of PI.

## Introduction

Lung cancer remains at the forefront of cancer-related mortality, with adenocarcinoma as its most prevalent subtype^1^. Pleural invasion (PI), identified as tumour penetration beyond the elastic layer of the visceral pleura, is acknowledged as a negative prognostic factor for non-small-cell carcinoma (NSCLC). It is associated with a notable decline in survival rates^2^, increased instances of local recurrence^3^, and more widespread lymph node metastases^4^. The 8th edition of the TNM classification integrates visceral pleural invasion (VPI) and parietal pleural invasion as pathologic descriptors of T2 and T3, respectively^5^. Furthermore, PI serves as one of the indications for adjuvant chemotherapy postsurgical resection^6,7^. Essentially, the detection of PI can influence clinical staging, treatment decisions, and survival outcomes, underscoring the importance of its reliable identification prior to treatment decisions.

CT imaging holds substantial interest for radiologists and surgeons in the prediction of pleural invasion. Multiple studies have shown that a shorter distance between tumours and visceral pleura correlates with a higher occurrence of PI^8,9^. Additionally, tumours with pleural contact exhibit a higher PI rate than those without pleural contact^10,11^. Despite this, Zhao et al.^11^ reported that the incidence of PI in NSCLC with pleural contact was a mere 25.5%, indicating that pleural contact does not inherently imply PI. Conversely, prior research has shown that a blunt angle between tumours and pleura^12^, along with a more extended length of pleural contact^12,13^, could elevate the likelihood of PI. Tanaka et al.^12^ reached a diagnostic sensitivity and specificity of 63.6% and 88.1%, respectively, for PI in NSCLCs with pleural contact, when the cut-off value of contact length was 12 mm.

Moreover, CT features suggestive of tumour invasiveness, such as tumour size^14^ and tumour density^14,15^, have been reported to correlate with PI in NSCLCs with pleural contact. For instance, a previous study on pure ground glass opacities demonstrated that pleural retraction (which increases the incidence and degree of pleural contact) and a large diameter did not imply pleural invasion^15^. Iodine uptake derived from spectral CT images has also been found to be associated with localized invasiveness and the degrees of differentiation in NSCLCs^16,17^. For instance, Shimamoto et al.^16^ discovered that iodine-related attenuation of NSCLCs with a diameter less than 3 cm at the arterial phase was an independent predictor of tumour invasiveness, determined when tumours exhibited at least one of lymphatic, vascular, or pleural invasion. Furthermore, Li et al.^17^ found a correlation between lower iodine concentration (IC) at the venous phase and poorly differentiated lung cancers. Deng et al.^8^ reported a higher incidence of PI in poorly differentiated lung cancers than in moderately and well-differentiated lung cancers. These results suggest that iodine uptake could indicate tumour invasiveness and increased occurrence of PI. However, the relationship between iodine uptake and pleural invasion remains unclear, as only a limited number of relevant studies have been conducted.

In the current study, the focus was to examine whether CT features suggestive of tumour invasiveness or iodine uptake serve as independent predictors for PI in adenocarcinomas (ACCs) with pleural contact.

## Materials and methods

### Ethical approval

The Ethics Committee of Zhongshan Hospital Affiliated with Xiamen University approved this retrospective cohort study and waived the requirement for patient informed consent. Our study adhered to the tenets of the Declaration of Helsinki.All methods were carried out in accordance with the relevant guidelines and regulations.

### Participants

**Figure 1:**
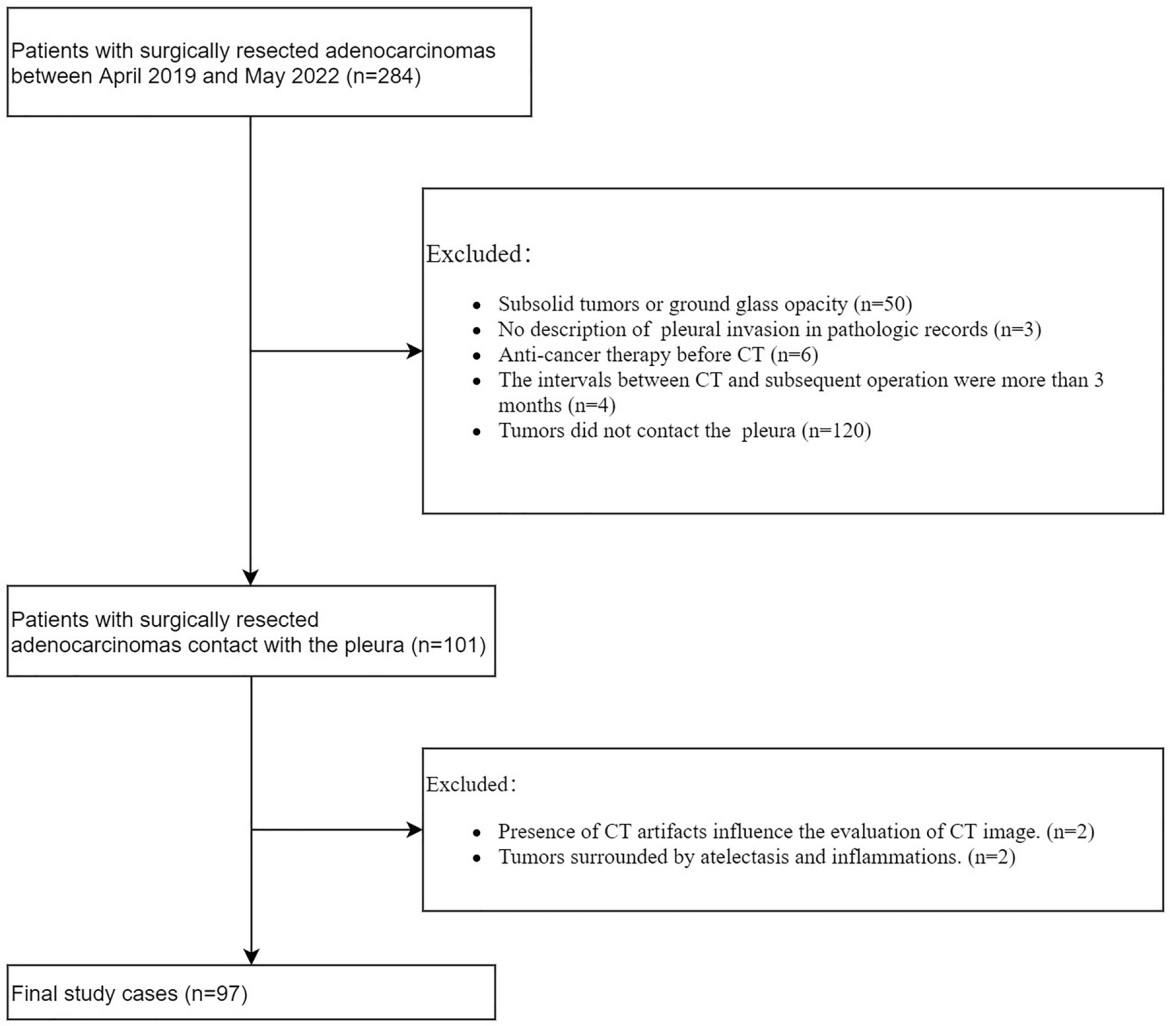
Flow diagram of patient inclusion and exclusion.

Out of 284 consecutive patients with pathologically confirmed ACCs by resected specimens between April 2019 and May 2022, 97 patients with solid tumours were included in this study (Fig. 1). The inclusion criteria were as follows: (a) purely solid tumours; (b) resected ACCs with a pathological record of pleural invasion description; (c) no prior chemotherapy or radiotherapy before CT; (d) intervals of less than three months between CT and subsequent operation; and (e) tumours in contact with either the shifted or nonshifted pleura. The exclusion criteria included: (a) CT artefact that interfered with lesion measurement; and (b) tumours surrounded by atelectasis and inflammation.

### CT imaging

All study participants underwent a spectral CT scan (IQON, Philips Health care, Germany) following a consistent protocol prior to tumour resection. Approximately 55–60 ml of iodinated contrast agent (Iopromide, 300 mg/ml, Bayer Health care, Guangzhou, China) bolus was administered at a rate of 3 mL/s via the antecubital vein, facilitated by a high-pressure injector (OptiVantage, Liebel-Flarsheim Company, Cincinnati OH, USA), followed by 30 ml of saline solution delivered at 3 mL/s. Automatic bolus tracking within the ROI, positioned on the descending aorta at the tracheal bifurcation level, was used for the arterial enhancing phase. The threshold attenuation of the ROI was set to 100 HU, which, upon being reached, served as the trigger for automatically initiating the scan. The venous phase scan commenced 25 s after the arterial phase scan was completed. The scanning boundary, encompassing the thoracic inlet to the adrenal gland, remained consistent for both plain and contrast scans. The CT scan parameters were set as follows: tube voltage, 120 kVp; matrix, 512 × 512; collimation, 64 × 0.625 mm; slice thickness, 2 mm; increment, 2 mm; pitch, 0.891; rotation time, 0.5 s. A DoseRight index (DRI) (automatic exposure control technique, Philips Health care) of 17 was set for medium-sized patients and the assumed reference current was 73 mA, with a DRI of 21 set for liver area. The mean volume CT dose index (CTDIvol) for the patients in this study was 21.3 mGy (range, 18.6–22.8 mGy). The raw data from the arterial and venous phases were relayed to an advanced workstation (IntelliSpace Portal V10.1.1.21740, Philips Corp, Netherlands) and were reconstructed to create multiple planar images and iodine overlay images with a section thickness and slice interval of 0.625 mm each.

### Imaging evaluation on CT

The multiplanar CT images were independently scrutinized within the same week by two experienced radiologists, Ying-Dong Chen and Qian-wen Huang, who had eight and five years of experience in pulmonary radiologic diagnosis, respectively, and were unaware of pathologic reports indicating PI.

**Figure 2:**
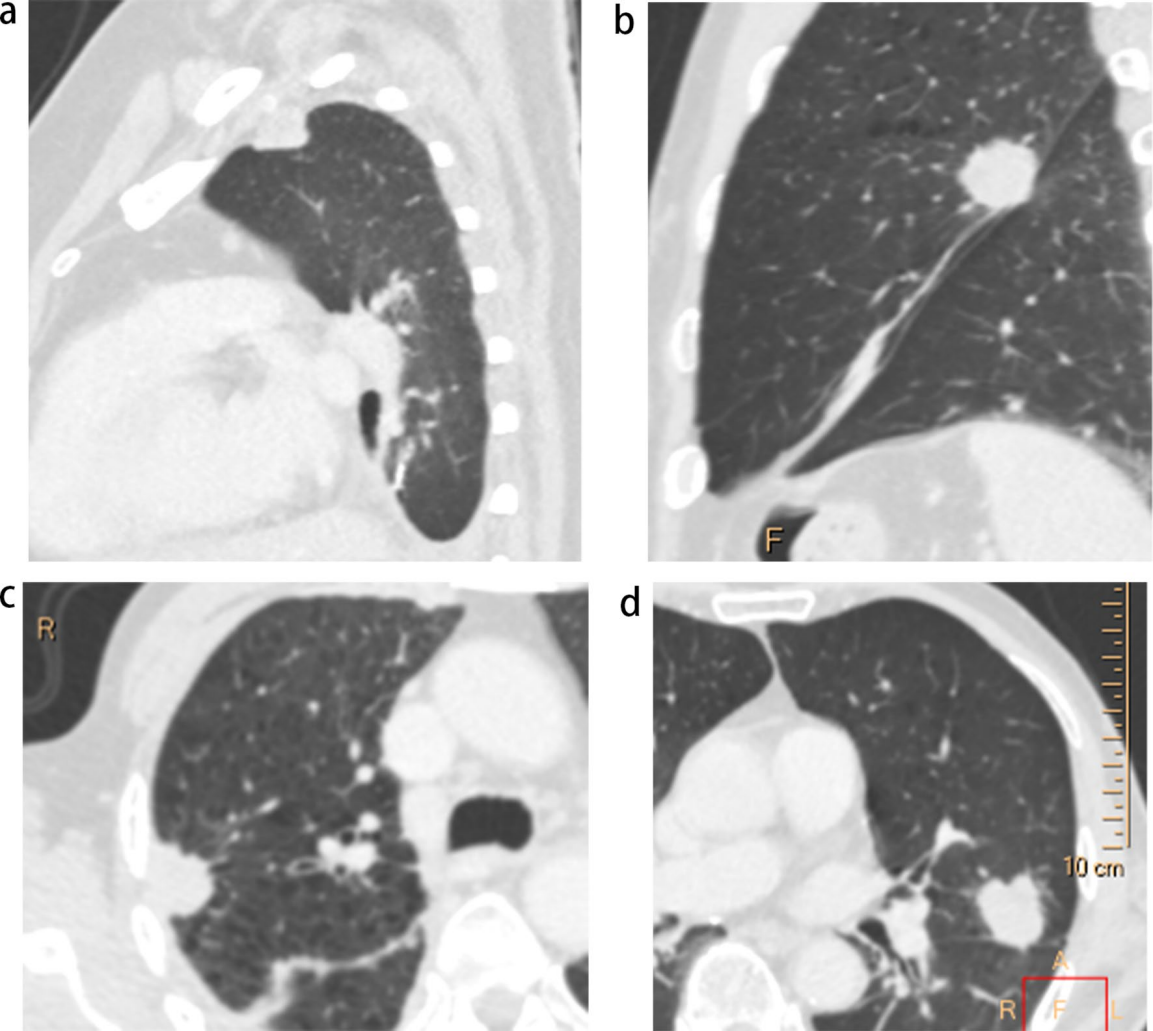
Representative CT images of the pleura being pushed and blunt angle. (**a**) Tumor straddled the costal pleura and partly occupied the extrapleural space. (**b**) Tumor pushed the interlobar pleura in the opposite direction. (**c**) Blunt angle between tumour and costal pleura (ventral side). (**d**) Sharp angle between tumour and interlobar pleura.

**Figure 3:**
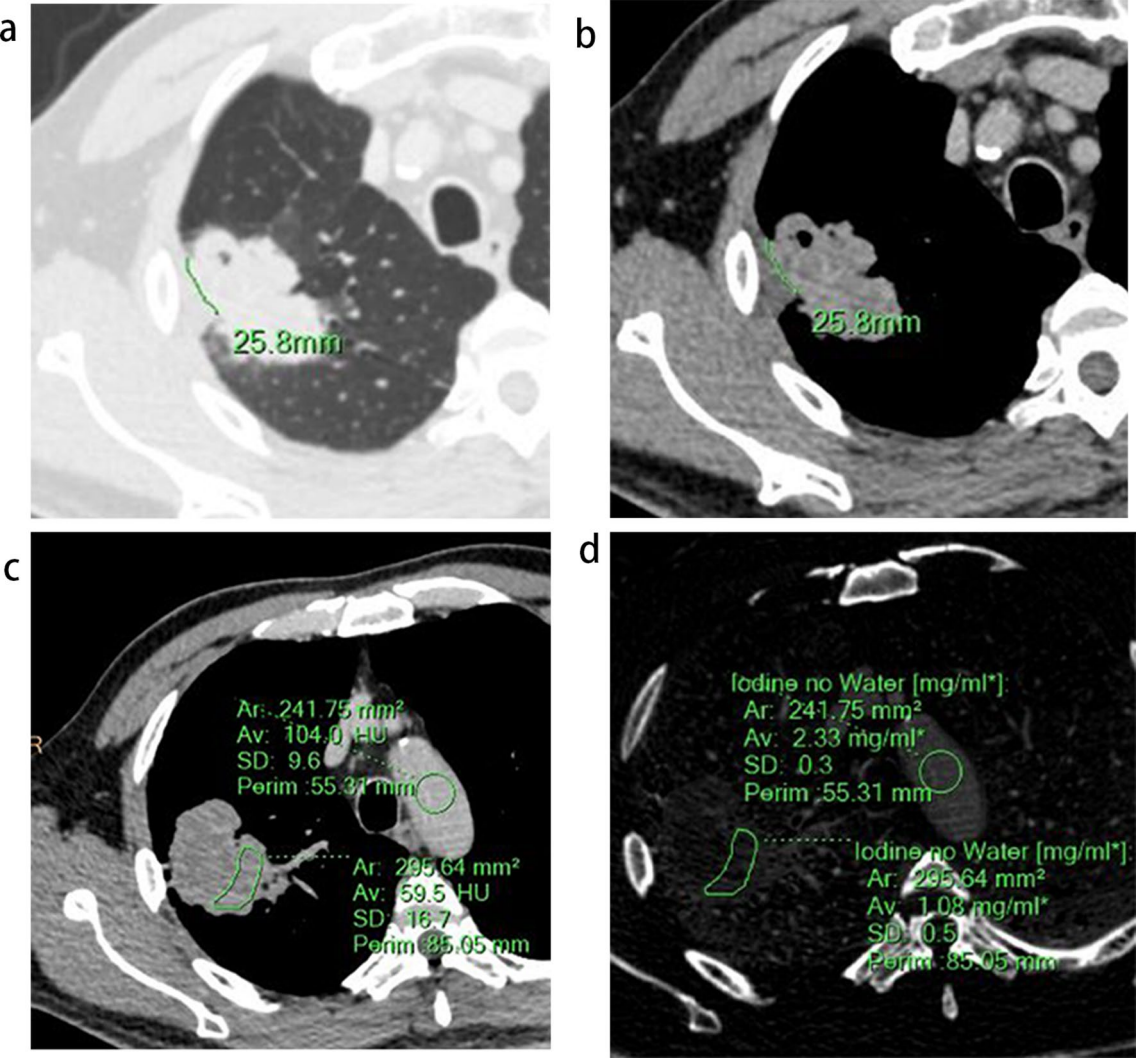
Measurement of contact length and Iodine uptake. (**a**, **b**) the largest interface length between the tumor and pleura on multiple planes was measured by manually outlining the curve of interface. (**c**) a region of interest (ROI) drawn freehand to cover at least 25% of the nonnecrotic lesion was placed at the venous phases, and a circular ROI was placed in the center of aorta on the same slice. (**d**) Iodine concentration of the lesion and aorta were derived from iodine overlay image.

Assessment of the following CT features took place on lung windows (window level: − 600 HU, window width: 1600 HU) at the venous phase: location, morphology (regular, lobulated, or irregular), and the presence of pleural retraction, bronchial occlusion, spiculation sign, and vacuole sign. Pleural retraction was defined as the retraction of the visceral pleura towards the tumour^8^, while bronchial occlusion was identified when bronchi were occluded within the tumour or at the proximal margin. The spiculation sign was denoted by unbranched stripe shadows originating from the tumour margins and extending into the pulmonary parenchyma. The pleura being pushed was diagnosed when the tumour pushed or straddled the pleura (Fig. 2a,b). The vacuole sign was considered present when a round lucency < 5 mm was found within the tumour. Tumour size was documented as the greatest diameter, while contact length with the pleura was measured by manually outlining the curve of the largest interface length between the tumour and pleura on multiple planes (Fig. 3a,b)^18^.The blunt angle between tumours and pleura was identified when a blunt angle occurred on either side on the slice with maximum contact length (Fig. 2c,d). On the slice with maximum diameter (mediastinal windows, window level: 40 HU, window width: 350 HU), a region of interest (ROI) drawn freehand to cover at least 25% of the nonnecrotic lesion was placed at both the arterial and venous phases (Fig. 3c,d), ensuring that areas of necrosis, large vessels, and calcifications were avoided^19^. The position and size of the ROIs for both phases were drawn as consistently as possible. Iodine concentration (IC) at arterial (ICA) and IC at venous phase (ICV) in the ROI were measured by converting conventional enhanced images to iodine overlay images, along with IC in the centre of the aorta on the same slice at both phases. Normalized IC at arterial (NICA) and at venous phase (NICV) was calculated by the quotient value of IC of lesions divided by IC of the aorta.

Any disagreements during image evaluations prompted a discussion between observers to reach consensus, and numerical values obtained from measurement were averaged.

### Pathologic evaluation

Pathologic results were procured from the pathological records of the institution. The pathologic slices were stained using heamatoxylin-eosin, or additional Verhoeff–Van Gieson staining if needed. An experienced pathologist, with seven years of experience in thoracic pathology and blind to clinical data, categorized PI according to the 8th TNM staging system into two groups: PI −, indicating no tumour invasion of the visceral pleura, and PI +, indicating tumour invasion beyond the elastic layer of the visceral pleura.

### Statistical analysis

For continuous variables, normal distribution was tested using the Kolmogorov‒Smirnov test. Normally distributed data are expressed as the means and standard deviations for continuous variables, or as medians and interquartile ranges (IQRs) for nonnormally distributed data. Categorical variables are described as frequencies with percentages. Size and contact length were dichotomized with cut-off values of 18 mm and 10 mm, respectively. To assess differences in CT features between ACCs with PI + and PI −, the Mann‒Whitney test and the independent sample test were used for nonnormally distributed and normally distributed continuous data, respectively. The χ^2^ test or Fisher's exact test was used for categorical data. Categorical variables with statistical significance were included as input variables to perform binomial logistic regression analysis with a forwards Selection (likelihood ratio) model in which variables were removed by a *P* value > 0.1. Finally, accuracy analyses and receiver operating characteristic curves were constructed to analyse predictive efficiency. All data analyses were conducted using the SPSS statistical software package (version R26; IBM Corp). A two-tailed *p* value < 0.05 was considered statistically significant.

## Results

### Patient and tumour characteristics

The cohort consisted of 97 consecutive patients with a mean age of 61.8 years ± 10.0; 48 of whom were females. The patient population included 24 out of 97 patients (24.7%) who were either smokers or ex-smokers, and 8 out of 97 patients (8.2%) displayed a previous history or familial history of malignant tumours. tumourThe median size of the tumours was 25.0 mm. PI was detected in 60 out of 97 patients (61.9%). Additional details are provided in Table 1.

**Table 1:** Clinical characteristics of 97 patients with solid adenocarcinomas.

Variables	Datum
No. patients	97
Age, mean ± SD^†^, y	61.8 ± 10.0
Sex	
Man	49
Women	48
Size, median ± IQR^‡^, mm	24 ± 16.4
Smoking history	
No	24
Yes	73
History of malignancy	
No	89
Yes	8
Tumour location	
Left upper lobe	22
Left lower lobe	17
Right upper lobe	25
Right middle lobe	10
Right lower lobe	23
Pleural invasion	
No	37
Yes	60

^†^Standard deviation.

^‡^Interquartile range.

### CT features, iodine uptake and their association with the PI

Univariate analyses were conducted to identify differences in clinical and CT features between ACCs with PI and those without. Compared to the PI − group, the PI + group exhibited a larger size (27.0 mm vs. 21.1 mm, *p *= 0.01), contact length (19.9 mm vs. 9.1 mm, *p *= 0.00), spiculation sign (56.7% vs. 32.4%, *p *= 0.02), bronchial occlusion (46.7% vs. 21.6%, *p *= 0.01), vacuole sign (18.3% vs. 0%, *p *= 0.00), contact length > 10 mm (76.7% vs. 40.5%, *p *= 0.00), and pleura being pushed (46.3% vs. 21.6%, *p *= 0.03). However, no significant differences were identified regarding age, sex, morphology, pleural retraction, or blunt angle between the PI + group and the PI − group (all *P *> 0.05) (Table 2).

**Table 2:** Comparison of CT features and iodine uptake between solid adenocarcinomas with or without pleural invasion.

Variables	Total, N (%)	PI^†^ +, N (%)	PI −, N (%)	*P* value
Age, mean ± SD^‡^	61.8 ± 10.0	62.9 ± 9.9	60.0 ± 10.0	0.66
Sex				0.26
Male	49 (50.5)	33 (55.0)	16 (43.2)	
Female	48 (49.5)	27 (45.0)	21 (56.8)	
Size				0.01*
Median ± IQR^§^, mm	24 ± 16.4	27.0 ± 18.2	21.1 ± 17.7	
Morphology				0.10
Regular	32 (22.7)	12 (20)	10 (27.0)	
Lobulated	68 (70.1)	46 (76.7)	22 (59.5)	
Irregular	7 (7.2)	2 (28.6)	5 (13.5)	
Spiculation sign				0.02*
No	51 (52.6)	26 (43.3)	25 (67.6)	
Yes	46 (47.4)	34 (56.7)	12 (32.7)	
Bronchial obstruction				0.20
No	72 (78.4)	44 (73.3)	32 (86.5)	
Yes	21 (21.6)	16 (26.7)	5 (13.5)	
Vacuole sign				0.01*
No	86 (88.7)	49 (87.7)	37 (100)	
Yes	11 (11.3)	11 (18.3)	0 (0)	
Pleural retraction				0.51
No	30 (30.9)	20 (33.3)	10 (27.0)	
Yes	67 (69.1)	40 (66.7)	27 (73.0)	
Pleura being pushed				0.03*
No	63 (64.9)	34 (56.7)	29 (78.4)	
Yes	34 (35.1)	26 (46.3)	8 (21.6)	
Contact length				0.00*
< 10 mm	38 (37.1)	14 (23.3)	22 (59.5)	
> 10 mm	61 (62.9)	46 (76.7)	15 (40.5)	
Median ± IQR, mm	15.3 ± 16.9	19.9 ± 15.3	9.1 ± 11.9	0.00*
Blunt angle				0.20
No	21 (21.6)	16 (26.7)	5 (13.5)	
Yes	76 (78.4)	44 (73.3)	32 (86.5)	
Iodine uptake^¶^				
ICA, median ± IQR, mg/ml	1.51 ± 0.65	1.56 ± 0.95	0.58 ± 0.24	0.21
NICA, median ± IQR	0.16 ± 0.08	0.16 ± 0.11	0.15 ± 0.65	0.39
ICV, median ± IQR, mg/ml	1.62 ± 0.65	1.64 ± 0.97	1.53 ± 0.60	0.30
NICV, mean ± SD	0.52 ± 0.20	0.58 ± 0.24	0.49 ± 0.16	0.06

^†^*PI* Pleural invasion.

^‡^Standard deviation.

^§^Interquartile range.

^¶^*ICA* and *NICA* Iodine Concentration and normalized Iodine Concentration at arterial phase. *ICV* and *NICV* Iodine Concentration and normalized Iodine Concentration at venous phase.

*p < 0.05.

The mean values of ICA and ICV were 1.51 and 1.62 mg/ml, respectively. No significant differences in ICA, ICV, NICA, NICV were discerned between the PI + group and the PI − group (Table 2).

A binomial logistic regression model was built using statistically significant CT features from the univariate analyses, which included contact length > 10 mm, spiculation sign, pleura being pushed, vacuole sign, bronchial occlusion, and size > 18 mm. As illustrated in Table 3, contact length > 10 mm (OR 4.80, 95% CI 1.92, 11.99, *p *= 0.001), and spiculation sign (OR 2.71, 95% CI 1.08, 6.79, *p *= 0.033) were eventually incorporated into the regression model, indicating that contact length > 10 mm and spiculation sign were independent predictors of PI.

**Table 3:** Multivariate analysis of potential predictor for pleural invasion in solid adenocarcinomas with pleural contact.

Variables	OR^†^	CI^‡^	*P* value
Contact length > 10 mm	4.80	1.92–11.98	0.00*
Spiculation sign	2.71	1.08–6.79	0.03*

^†^*OR* odds ratio.

^‡^*CI* Confidence interval.

*p < 0.05.

### Diagnostic efficiency for PI

**Figure 4:**
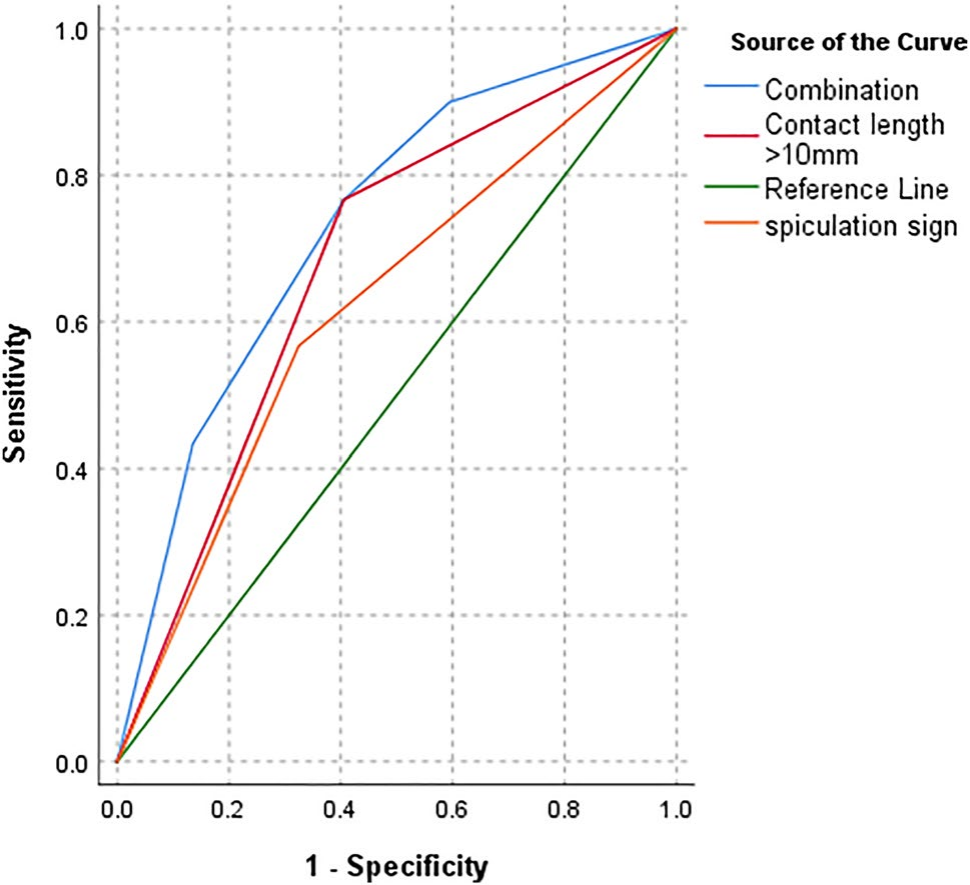
Receiver operating characteristic (ROC) curves for predicting Pleural invasion. The area under curve (AUC) of contact length > 10 mm (purple line), spiculation sign (orange line) and combination of two indicators (blue line) were 0.68, 0.62, 0.73, respectively.

Table 4 demonstrates that the sensitivity, specificity, positive predictive value (PPV), negative predictive value (NPV) and accuracy of prediction for PI via contact length > 10 mm itself were 76.7%, 59.5%, 75.4%, 61.1%, and 70.1%, respectively; and 56.7%, 67.6%, 73.9%, 49.0%, and 60.8%, for spiculation sign, respectively. However, a higher sensitivity (90.0%) was achieved by the combination of the above two CT features, with a specificity, PPV, NPV, and accuracy of 40.5%, 71.1%, 71.4%, and 71.1%, respectively. ROC curves revealed that the areas under the curve (AUC) of contact length > 10 mm, spiculation sign, and the combination of two indicators were 0.68, 0.62, and 0.73, respectively (Fig. 4). Therefore, the combination of contact length > 10 mm and spiculation sign enhanced the diagnostic performance of PI.

**Table 4:** Accuracy analysis of significant predictors for pleural invasion.

Predictors	Sensitivity, %	Specificity, %	PPV^†^, %	NPV^‡^, %	Accuracy, %
Contact length > 10 mm	76.7	59.5	75.4	61.1	70.1
Spiculation sign	56.7	67.6	73.9	49.0	60.8
Combination^§^	90	40.5	71.1	71.4	71.1

^†^*PPV* Positive predictive value.

^‡^*NPV* Negative predictive value.

^§^Combination of contact length and spiculation sign.

## Discussion

The present study was undertaken to explore whether CT features suggestive of tumour invasiveness or iodine uptake served as independent predictors for pleural invasion (PI) in adenocarcinomas (ACCs) with pleural contact. The analysis ultimately identified contact length > 10 mm and spiculation sign as independent predictors of PI, based on the results of the multivariate analysis. Remarkably, a sensitivity of 90.0% was achieved in predicting PI by the combination of these two indicators. Surprisingly, iodine uptake exhibited no correlation with PI.

Contact length > 10 mm was anticipated to be associated with PI in ACCs with pleural contact. First, the presence of significant differences was affirmed between the PI + group and PI − group with respect to contact length and contact length > 10 mm. Subsequently, contact length > 10 mm was established to be an independent predictor for PI. Reinforcing these findings, Tanaka et al.^12^ ratified that contact length was a significant independent indicator for PI in solid ACCs with pleural contact and size < 30 mm, and was larger in tumours with PI than without (19 mm vs. 5 mm). Ebara et al.^18^ corroborated that solid peripheral lung cancers with pleural contact demonstrated a larger interface length between tumours and pleura (15.4 mm vs. 12.0 mm) when PI was present. Although Heidinger et al.^20^ observed that pleural contact varied statistically between solid lung cancers with PI and those without, it was not recognized as an independent factor for predicting PI, a distinction from the present study. However, this study measured the curvilinear length of the interface between tumours and pleura as contact length, which could more accurately evaluate the contact length than the linear measurement employed by Heidinger et al., potentially leading to a discrepancy between the studies. Moreover, solid contact length was also reported as an independent predictor for PI in a study involving subsolid and pure-solid ACCs with pleural contact, with median values of 15.1 mm and 7.7 mm in the PI + group and PI − group, respectively^10^. A longer contact length might suggest a more extended period of tumour development, providing more pleural sites for invasive tumours to penetrate, thus increasing the probability of PI.

The spiculation sign, a known CT feature indicative of malignant lung neoplasms^21,22^, may suggest tumour invasiveness and a poorer prognosis in lung cancers to some extent. For instance, the 5-year survival rate of stage I NSCLCs with spiculation signs declined from 83.3 to 39.5%, compared with tumours with a normal margin^23^. Additionally, tumours exhibiting more invasiveness are likely to invade the visceral pleura more quickly and easily when the tumour abuts the pleura. This study identified the spiculation sign as a significant predictor for PI. In contrast, while Wang et al.^24^ reported that the spiculation sign significantly differed between stage I NSCLCs with PI and those without, with 26 out of 33 (78.8%) patients with the spiculation sign having PI^24^, and Deng et al.^8^ found that NSCLCs with PI showed a statistically higher rate of spiculation (86.8% vs. 45.6%), neither of these studies recognized the spiculation sign as an independent predictor for PI^8,24^. It should be noted that this study concentrated on lung cancers with pleural contact, which differs from lung cancers with nonspecific tumour-pleura relationships examined in other studies. It can be hypothesized that the impact of spiculation on tumours with pleural contact may be more pronounced than that on those with pleural tags or proximity to the pleura, as the pleura is more easily affected by tumours with pleural contact. In conclusion, the spiculation sign is significantly correlated with PI in ACCs with pleural contact.

Tanaka et al.^12^ utilized a 12 mm cut-off value for contact length and discovered that the diagnostic sensitivity for PI was 63.6%, a figure lower than the 76.7% observed in this study. Furthermore, this study derived a higher sensitivity (90.0%) by integrating the spiculation sign and contact length > 10 mm for predicting PI, in comparison to sensitivity derived by employing a single indicator. Therefore, the spiculation sign may enhance the diagnostic performance of PI in ACCs with pleural contact, a fact not previously clarified.

Spectral CT has recently been widely employed in respiratory imaging, facilitating the quantification of iodine uptake in lung cancers through the application of iodine-overlay images^25^. Iodine uptake has been reported to correlate with the degree of differentiation^26^ and angiogenesis^27^, both of which are related to tumour invasiveness. Li et al. showed a positive correlation between IC at the arterial, venous, and delayed phases with microvessel densities of lung cancers. They also demonstrated that IC at the venous phase was statistically higher in poorly differentiated lung cancers compared to moderately or highly differentiated tumours^17^. However, scant literature addresses the relationship between PI and iodine uptake. To the authors' knowledge, only Doai et al.^28^ reported that IC at the delayed phase did not correlate with PI, a finding attributed to necrosis or degeneration of tumours. This study also determined that iodine uptake at the arterial and venous phases did not correlate with PI in ACCs with pleural contact. A plausible explanation for this observation may be that IC can be influenced by multiple complex factors, such as the degree of differentiation, the pathologic subtype, the size, and the presence of intratumoural necrosis. For instance, lower enhancement and iodine uptake can be seen in poorly differentiated, larger, and necrotic tumours, compared to highly differentiated, small, and nonnecrotic tumours^17^.

Several limitations are acknowledged regarding this study. First, the sample size of this study was small due to the stringent inclusion criteria, leading to a possible patient selection bias. However, two experienced radiologists were utilized to ensure the accuracy of the image evaluation. Second, we did not evaluate the difference in CT features between varying degrees of PI, as only a few cases demonstrated a higher degree of PI. Third, elastin staining was not applied to all specimens during the pathological evaluation of pleural invasion, which might influence the accuracy of pathological evaluation. Therefore, further investigations with larger sample sizes are necessary to validate the findings of this study.

In this study, we concluded that contact length > 10 mm and spiculation sign serve as independent predictors of PI in ACCs with pleural contact, and the combination of these two indicators could augment the diagnostic performance of PI. Moreover, with this study, we have established that iodine uptake exhibits no correlation with PI, even though it indicates tumour invasiveness.

## Data Availability

The data presented in this study are available on request from the corresponding author. The data are not publicly available due to privacy of patients.

## References

[1] Siegel RL, Miller KD, Jemal A (2019). Cancer statistics, 2019. CA Cancer J Clin.

[2] Jiang L (2015). The impact of visceral pleural invasion in node-negative non-small cell lung cancer: a systematic review and meta-analysis. Chest.

[3] Dziedzic DA, Rudzinski P, Langfort R, Orlowski T (2016). Risk factors for local and distant recurrence after surgical treatment in patients with non-small-cell lung cancer. Clin Lung Cancer.

[4] Kudo Y (2012). Impact of visceral pleural invasion on the survival of patients with non-small cell lung cancer. Lung Cancer.

[5] Feng S, Yang S (2019). The new 8th TNM staging system of lung cancer and its potential imaging interpretation pitfalls and limitations with CT image demonstrations. Diag Interv Radiol.

[6] Kuriyama S (2022). Using CT to evaluate mediastinal great vein invasion by thymic epithelial tumours: measurement of the interface between the tumour and neighboring structures. Eur Radiol.

[7] Tsutani Y (2022). Adjuvant chemotherapy for high-risk pathologic stage I non-small cell lung cancer. Ann Thorac Surg.

[8] Deng H-Y (2018). Novel biologic factors correlated to visceral pleural invasion in early-stage non-small cell lung cancer less than 3 cm. J Thorac Dis.

[9] Qi L (2016). Multivariate analysis of pleural invasion of peripheral non-small cell lung cancer-based computed tomography features. J Comput Assist Tomogr.

[10] Shi J (2021). The combination of computed tomography features and circulating tumour cells increases the surgical prediction of visceral pleural invasion in clinical T1N0M0 lung adenocarcinoma. Transl Lung Cancer Res.

[11] Zhao L-L (2016). Visceral pleural invasion in lung adenocarcinoma ≤ 3 cm with ground-glass opacity: a clinical, pathological and radiological study. J Thorac Dis.

[12] Tanaka T (2015). Predicting pleural invasion using HRCT and 18F-FDG PET/CT in lung adenocarcinoma with pleural contact. Ann Nucl Med.

[13] Glazer H (1985). Pleural and chest wall invasion in bronchogenic carcinoma: CT evaluation. Radiology.

[14] Ahn SY (2017). Predictive CT features of visceral pleural invasion by T1-sized peripheral pulmonary adenocarcinomas manifesting as subsolid nodules, AJR. Am J Roentgenol.

[15] Wang Z, Zhu W, Lu Z, Li W, Shi J (2021). Invasive adenocarcinoma manifesting as pure ground glass nodule with different size: radiological characteristics differ while prognosis remains the same. Transl Cancer Res.

[16] Shimamoto H, Iwano S, Umakoshi H, Kawaguchi K, Naganawa S (2016). Evaluation of locoregional invasiveness of small-sized non-small cell lung cancers by enhanced dual-energy computed tomography. Cancer Imaging.

[17] Li Q (2020). Spectral CT in lung cancer: usefulness of iodine concentration for evaluation of tumour angiogenesis and prognosis, AJR. Am J Roentgenol.

[18] Ebara K (2015). Pleural invasion by peripheral lung cancer: prediction with three dimensional CT. Acad Radiol.

[19] Kiessling F (2004). Perfusion CT in patients with advanced bronchial carcinomas: a novel chance for characterization and treatment monitoring?. Eur Radiol.

[20] Heidinger BH (2019). Visceral pleural invasion in pulmonary adenocarcinoma: differences in CT patterns between solid and subsolid cancers. Radiol Cardiothorac Imaging.

[21] Tian K, Li Z, Qin L (2022). Detection of CEA and ProGRP levels in BALF of patients with peripheral lung cancer and their relationship with CT signs. Biomed Res Int.

[22] Snoeckx A (2018). Evaluation of the solitary pulmonary nodule: size matters, but do not ignore the power of morphology. Insights Imaging.

[23] Ma J (2017). Relationship between computed tomography morphology and prognosis of patients with stage I non-small cell lung cancer. Onco Targets Ther.

[24] Wang Y (2023). Multivariate analysis based on the maximum standard unit value of (18)F-fluorodeoxyglucose positron emission tomography/computed tomography and computed tomography features for preoperative predicting of visceral pleural invasion in patients with subpleural clinical stage IA peripheral lung adenocarcinoma. Diagn Interv Radiol.

[25] Lennartz S (2019). Dual-energy CT-derived iodine maps: use in assessing pleural carcinomatosis. Radiology.

[26] Lin L-Y (2018). Correlation between dual-energy spectral CT imaging parameters and pathological grades of non-small cell lung cancer. Clin Radiol.

[27] Chen X-H (2017). Spectral computed tomography in advanced gastric cancer: Can iodine concentration non-invasively assess angiogenesis?. World J Gastroenterol.

[28] Doai M (2022). Quantitative evaluation of iodine and fat using dual-energy CT for assessments of the tumour aggressiveness in lung cancer. Egypt J Radiol Nucl Med.

